# Thioredoxin interacting protein protects mice from fasting induced liver steatosis by activating ER stress and its downstream signaling pathways

**DOI:** 10.1038/s41598-022-08791-z

**Published:** 2022-03-21

**Authors:** Hiroyuki Miyahara, Kosei Hasegawa, Masato Yashiro, Toshiaki Ohara, Masayoshi Fujisawa, Teizo Yoshimura, Akihiro Matsukawa, Hirokazu Tsukahara

**Affiliations:** 1 Department of Pediatrics, Okayama University Hospital, 2-5-1 Shikata-cho, Kita-ku, Okayama, 700-8558 Japan; 2 Department of Pathology and Experimental Medicine, Okayama University Graduate School of Medicine, Dentistry and Pharmaceutical Sciences, Okayama, Japan; 3 Department of Pediatrics, Okayama University Graduate School of Medicine, Dentistry and Pharmaceutical Sciences, Okayama, Japan

**Keywords:** Metabolic syndrome, Chaperones, Endoplasmic reticulum

## Abstract

Under normal conditions, fasting results in decreased protein disulfide isomerase (PDI) activity and accumulation of unfolded proteins, leading to the subsequent activation of the unfolded protein response (UPR)/autophagy signaling pathway to eliminate damaged mitochondria. Fasting also induces upregulation of thioredoxin-interacting protein (TXNIP) expression and mice deficient of this protein (TXNIP-KO mice) was shown to develop severe hypoglycemia, hyperlipidemia and liver steatosis (LS). In the present study, we aimed to determine the role of TXNIP in fasting-induced LS by using male TXNIP-KO mice that developed LS without severe hypoglycemia. In TXNIP-KO mice, fasting induced severe microvesicular LS. Examinations by transmission electron microscopy revealed mitochondria with smaller size and deformities and the presence of few autophagosomes. The expression of β-oxidation-associated genes remained at the same level and the level of LC3-II was low. PDI activity level stayed at the original level and the levels of p-IRE1 and X-box binding protein 1 spliced form (sXBP1) were lower. Interestingly, treatment of TXNIP-KO mice with bacitracin, a PDI inhibitor, restored the level of LC3-II after fasting. These results suggest that TXNIP regulates PDI activity and subsequent activation of the UPR/autophagy pathway and plays a protective role in fasting-induced LS.

## Introduction

The liver plays a central role in maintaining systemic lipid homeostasis during fasting and feeding cycles. Under physiological conditions, fasting causes an energy source shift from glucose (Glu) to lipid, and oxidation of hepatic free fatty acids (FAs) supplies energy for Glu production^1^. Dysregulation of enzymes involved in hepatic FA β-oxidation and/or FA synthesis leads to liver steatosis (LS) or fatty liver^2,3^. During fasting, mitochondria is damaged by enhanced generation of mitochondrial reactive oxygen species due to the reduction in available energy sources; therefore, clearing damaged mitochondria is important to promote β-oxidation^4,5^.

Endoplasmic reticulum (ER) stress and its downstream signaling pathways, including the unfolded protein response (UPR) and autophagy, are essential for clearing damaged mitochondria^6^. During fasting, the activity of protein disulfide isomerase (PDI), that acts as a molecular chaperone, decreases and unfolded proteins accumulate in the ER, inducing ER stress and triggering the UPR^6–9^. ER stress is transmitted when accumulated unfolded proteins in the ER activate three sensors, inositol-requiring enzyme 1 (IRE1), activating transcription factor 6 (ATF6), and PKR-like endoplasmic reticulum kinase (PERK)^10–14^. Microtubule-associated protein light chain 3 (LC3) is activated after ER stress via a PERK- or IRE1-mediated pathway and plays an essential role in autophagy^11,15^. Damaged mitochondria are subsequently removed by autophagy.

Many metabolic diseases caused by dysfunction of mitochondrial β-oxidation-related enzymes show Reye-like syndrome^16,17^, and impairment in mitochondrial autophagy has been implicated as a cause of Reye-like syndrome^18^. Reye-like syndrome is characterized by abrupt onset, hypoglycemia, LS, and hyperlipidemia in the fasted state^16,19^. To date, the prognosis of these diseases remains dismal, and precise molecular mechanisms remain to be fully elucidated.

Mitochondrial disturbance is also a cause of fatty liver diseases. The two main etiologies of fatty liver are known as alcoholic liver disease (ALD) and nonalcoholic fatty liver disease (NAFLD). ALD is triggered by excessive alcohol intake and mainly explained by impaired citric acid (TCA) circuit^20^. In the case of NAFLD, excessive intakes of high fat diet or refined carbohydrates, and some genetic predispositions are also known as the cause^20^. Even though, mitochondrial deficiency and oxidative stress caused by mitochondrial dysfunction are common cause of NAFLD and ALD^20^.

Thioredoxin-interacting protein (TXNIP, also called thioredoxin binding protein 2 or vitamin D upregulated protein), was originally identified as a negative regulator of thioredoxin which is a common regulator of various redox reactions^21^. But, it also regulates the ER stress signaling and autophagy^22–24^, indicating that TXNIP can play a role in the UPR. In energy metabolism, TXNIP suppresses insulin secretion and plays a role in exacerbating glucotoxicity^22,25^.

The expression of TXNIP was markedly up-regulated during fasting in wild-type (WT) mice, whereas fasting caused fatal conditions in TXNIP knockout (TXNIP-KO) mice^19^, despite that they had no problems during the fed state. Interestingly, TXNIP-KO mice in response to fasting showed phenotypes of Reye-like syndrome, such as LS, hyperlipidemia, hypoglycemia, and hyperinsulinemia^19^. Mitochondrial deficiency was hypothesized to be an underlying cause of these phenotypes^19^; however, the exact molecular mechanisms involved in fasting-induced changes in TXNIP-KO mice have not been fully understood. We hypothesized that dysregulated ER stress, the UPR and its downstream autophagy signaling might be associated with the phenotypes observed in fasted TXNIP-KO mice.

In the present study, we aimed to define the TXNIP-mediated signaling pathways during fasting using TXNIP-KO mice. As previously reported, female mice developed severe phenotypes similar to those of Reye-like syndrome. Male mice also developed hyperlipidemia and LS but no severe hypoglycemia that could affect lipid metabolism and cause animal death. By using male TXNIP-KO mice, we here demonstrate evidence that TXNIP plays an important role in controlling the fasting-induced ER stress and activation of the UPR and autophagy in the liver in vivo and that TXNIP-KO mice develop LS because this controlling mechanism, especially the autophagy of damaged mitochondria, does not function well in the absence of TXNIP in the fasted state.

## Results

### More severe phenotypes in TXNIP-KO female mice than in male mice after fasting

**Figure 1:**
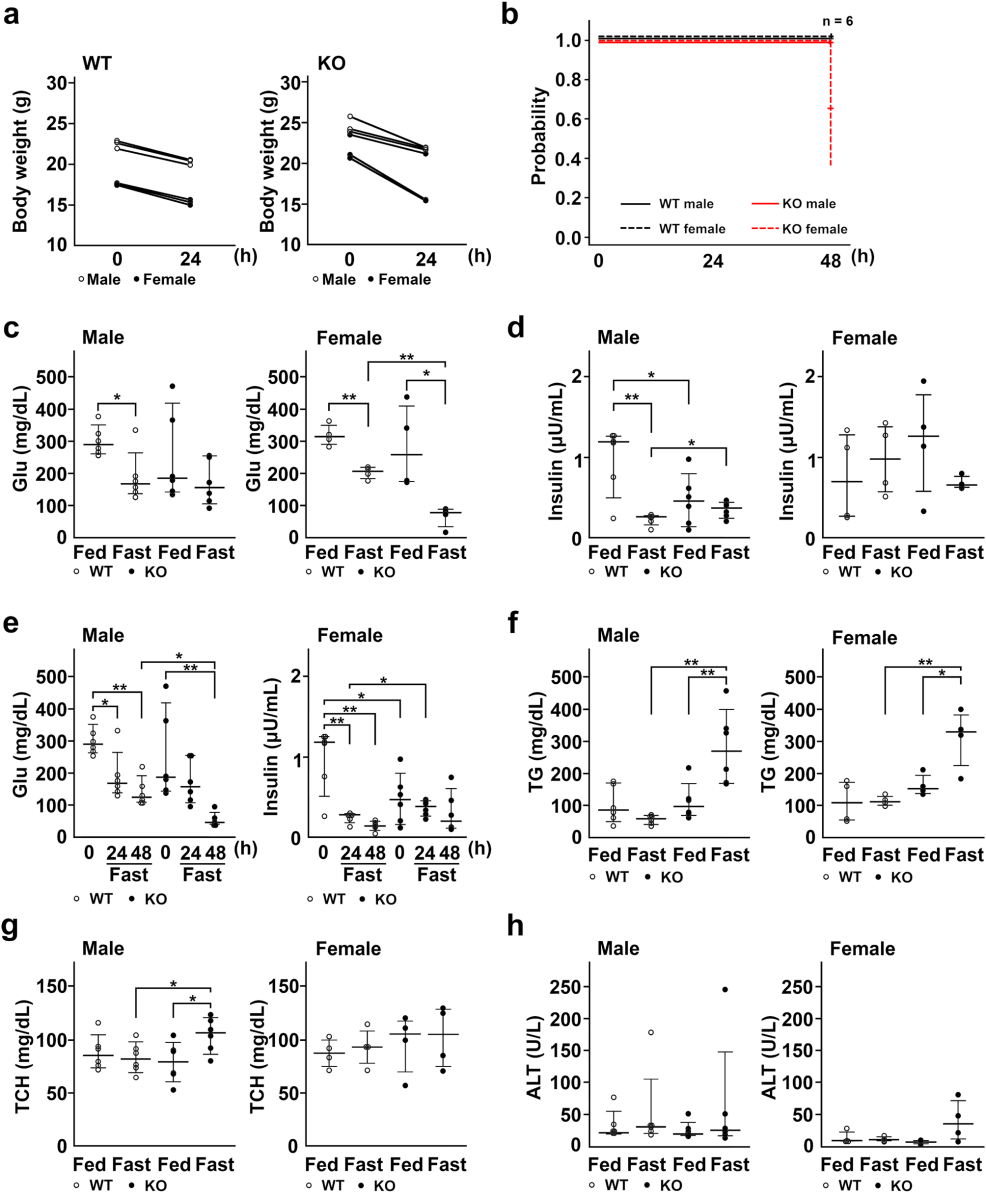
Hyperlipidemia occurs during fasting without changes in Glu levels in TXNIP-KO male mice. (**a**) Change of body weight was examined and shown by dot plots, revealing that WT and male TXNIP-KO mice lost weight after 24-h fasting and weight loss was more prominent in TXNIP-KO female mice (three mice per group). (**b**) TXNIP-KO female mice were vulnerable to fasting stress (six mice per group). (**c**–**h**) Laboratory data were collected from WT and TXNIP-KO mice in fed or fasted state and shown by dot plots; medians and 10–90 percentiles were also shown for each dot plot graph (six mice for male group and four mice for female group). (**c**,**d**) Despite Glu levels were statistically lower in female TXNIP-KO mice compared with female WT mice in fasted state, no statistical difference in Glu levels was found between WT and male TXNIP-KO mice in the fasted state. Serum insulin levels also showed no obvious difference between WT and TXNIP-KO mice (both in male and female mice), but serum insulin levels may be high in TXNIP-KO female mice when taking low blood Glu levels into account. (**e**) Serum Glu and insulin levels of TXNIP-KO male mice in fed state, or after 24-h or 48-h fasting. Although serum Glu level decreased to remarkably lower levels, serum insulin levels were relatively stable in TXNIP-KO mice compared with WT mice, indicating an impairment in the regulation of serum insulin level in response to serum glucose level in TXNIP-KO mice. (**f**,**g**) Although serum TG and TCH levels tended to be stable or decrease in WT mice, these values tended to increase in TXNIP-KO mice during fasting, and statistical differences were found in TG and TCH levels between WT and male TXNIP-KO mice and in TG between WT and female TXNIP-KO mice. (**h**) Serum ALT levels were measured. Although one mouse in WT and TXNIP-KO male mice showed highly increased serum ALT levels, serum ALT levels overall were stable in WT and TXNIP-KO male mice. In female mice, TXNIP-KO female mice tended to show increased serum ALT during fasted state. Reference values (mean ± SD) for male Glu, female Glu, male TG, female TG, male TCH, and female TCH were 306 ± 47 (mg/dL), 302 ± 33 (mg/dL), 104 ± 31 (mg/dL), 104 ± 33 (mg/dL), 91 ± 11 (mg/dL), 79 ± 19 (mg/dL) respectively. *p < 0.05, **p < 0.01. *WT* wild-type, *KO* knockout, *TXNIP* thioredoxin-interacting protein, *Glu* glucose, *TG* triglyceride, *FFA* free fatty acid, *TCH* total cholesterol, *ALT* alanine transaminase, *SD* standard deviation.

During this study, we first noted that female TXNIP-KO mice were more susceptible to fasting and developed more sever phenotypes. We therefore examined phenotypic differences in detail using male and female WT and TXNIP-KO mice after 24 h of fasting. As shown in Table 1, 4 of 6 TXNIP-KO female mice lost movement and showed hunched back posture. Two of 6 TXNIP-KO female mice also showed macroscopic hematuria (Table 1). By contrast, none of WT or TXNIP-KO male mice showed these phenotypes. TXNIP-KO female mice also lost more weight than WT male and female and TXNIP-KO male mice (Fig. 1a). The survival of mice during fasting was evaluated by the Kaplan–Meyer method. After 48 h of fasting, 4 out of 6 of female TXNIP-KO mice died, whereas none of WT or TXNIP-KO male mice died (Fig. 1b).

**Table 1:** Differences in phenotypes between TXNIP-KO male and female mice.

	Male				Female			
	WT 0	KO 0	WT 24	KO 24	WT 0	KO 0	WT 24	KO 24
Hematuria	0/6	0/6	0/6	0/6	0/6	0/6	0/6	2/6
Loss of movement	0/6	0/6	0/6	0/6	0/6	0/6	0/6	4/6
Hunched back posture	0/6	0/6	0/6	0/6	0/6	0/6	0/6	4/6

*WT0* wild-type mice before fasting, *KO0* knockout mice before fasting, *WT24* wild-type mice after 24-h fasting, *KO24* knockout mice after 24-h fasting.

We measured the levels of blood Glu, insulin, triglyceride (TG) and total cholesterol (TCH) before and after 24 h of fasting. In consistent with the previous report^19^, blood Glu level markedly decreased in fasted female TXNIP-KO mice (*p* < 0.001) (Fig. 1c), whereas there was no difference in blood Glu levels during the fasted state between WT and TXNIP-KO male mice (Fig. 1c). Since there was no significant difference in blood insulin levels in the fed and fasted state between male and female TXNIP-KO mice (Fig. 1d), the decreased Glu levels found in fasted female TXNIP-KO mice may be due to their higher sensitivity to insulin than male TXNIP-KO mice. Insulin levels were relatively maintained in TXNIP-KO male mice compared to WT mice after 48-h fasting (Fig. 1e), despite of their low blood Glu levels, suggesting a defect in the regulation of insulin secretion.

Blood TG level significantly (*p* = 0.001) increased in both male and female TXNIP-KO mice during fasting (Fig. 1f), similar to the previous report^19^. Blood TCH level was slightly but significantly higher (*p* = 0.035) in male TXNIP-KO mice, but this increase was not detected in female TXNIP-KO mice (Fig. 1g). Serum ALT level increased in one of WT and TXNIP-KO male mice, but was generally stable in both strains. In TXNIP-KO female mice, serum ALT levels slightly elevated after fasting but there was no statistical significance (Fig. 1h). Since severe hypoglycemia could affect lipid metabolism and also cause death, we used male TXNIP-KO mice for the rest of this study.

### Increased expression of TXNIP during the fasted state in WT mice

**Figure 2:**
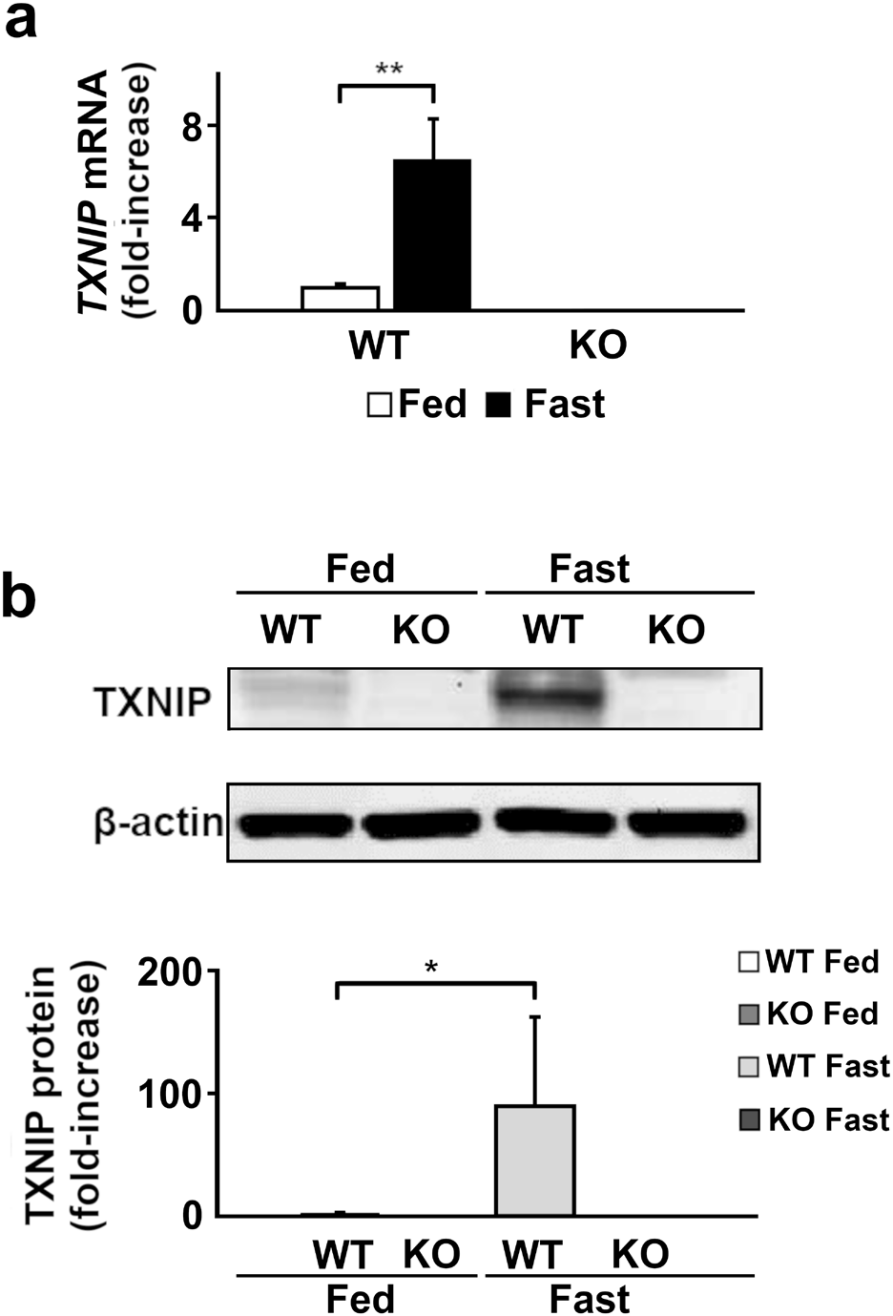
The expression of TXNIP is upregulated during fasting. The expression of TXNIP mRNA and protein in fed or fasted states was evaluated by RT-qPCR or western blotting. TXNIP mRNA (**a**) and protein (**b**) expression increased significantly in the fasted state in WT mice. TXNIP mRNA and protein expression was completely suppressed in TXNIP-KO mice in both states. *p < 0.05, **p < 0.01 (in each group, six mice were used for RT-qPCR, and ten were used for western blot analysis). *TXNIP* thioredoxin-interacting protein, *WT* wild-type, *KO* knockout mice.

To study the role of TXNIP in fasting-induced stress to the liver, we examined changes in the expression of TXNIP mRNA and protein in the liver of WT mice during fed and fasted states. In consistent with the previous report^23^, the expression of both TXNIP mRNA (*p* = 0.004) (Fig. 2a) and protein (*p* = 0.013) (Fig. 2b) increased in the fasted state. As expected, the expression of TXNIP was not detected in the TXNIP-KO mice at both mRNA and protein levels (Fig. 2a,b).

### Induction of hepatic microvesicular steatosis by fasting in TXNIP-KO mice

**Figure 3:**
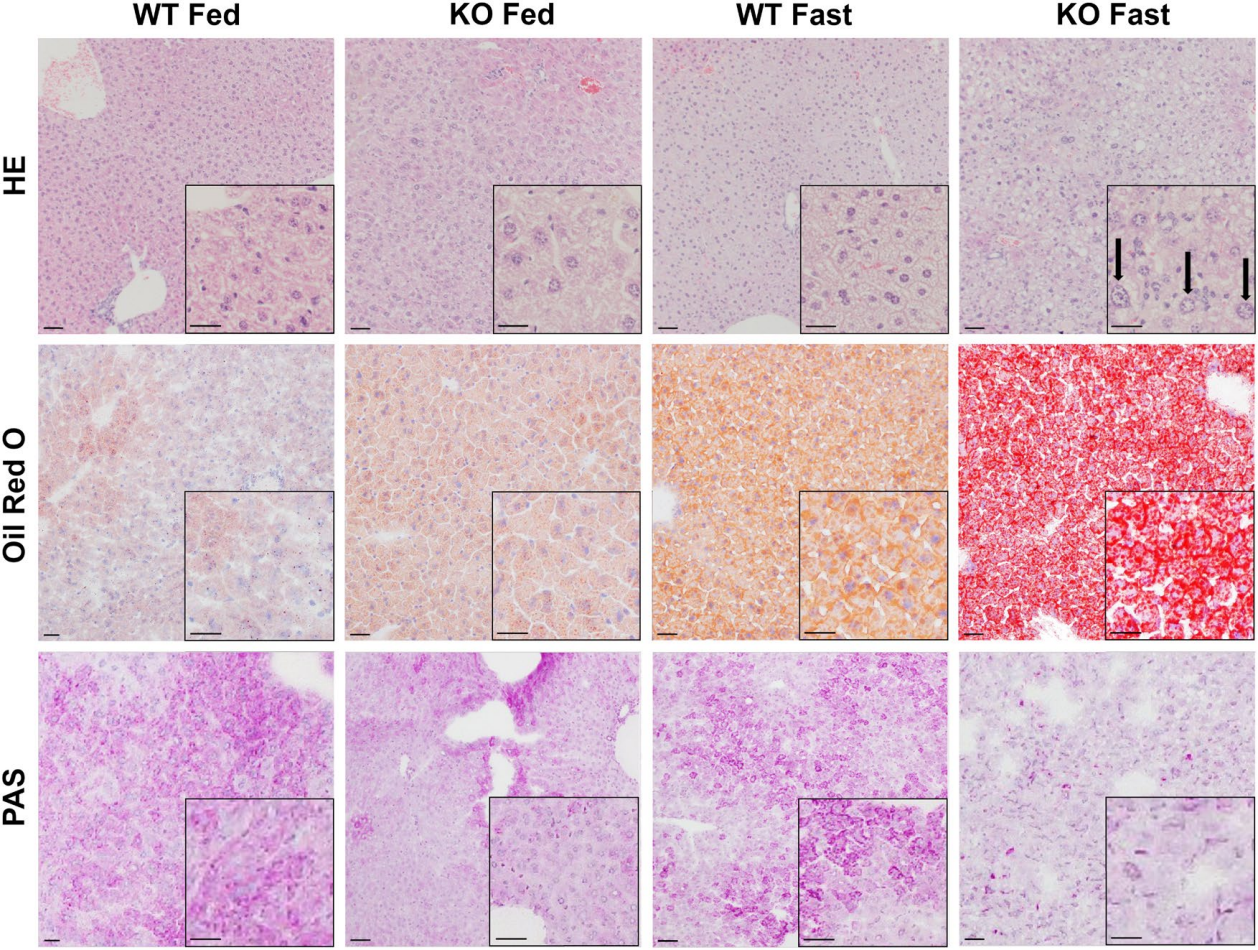
LS occurs during fasting in TXNIP-KO mice. LS during the fasted state in TXNIP-KO mice was verified by H&E, Oil Red O and PAS staining. Prominent hepatic microvesicular steatosis with enlarged nuclei (arrows) was observed in livers of TXNIP-KO mice in the fasted state by H&E staining, and accumulation of lipid droplets was detected by Oil Red O staining (scale bar = 50 μm). PAS staining showed depletion of glycogen in livers of fasted TXNIP-KO mice: depletion of glycogen was found even in the fed state. Representative photographs from each group are shown (six mice per group). *H*&*E* hematoxylin–eosin, *PAS* periodic acid Schiff, *TXNIP* thioredoxin-interacting protein, *WT* wild-type mice, *KO* knockout mice.

A previous report histologically demonstrated the induction of LS after fasting in TXNIP-KO mice^19^. Mitochondrial dysfunction has been proposed as the cause^19,26–28^. We also detected the development of microvesicular LS characterized by swollen hepatocytes with centrally located enlarged nuclei (arrows) and foamy-appearing cytoplasm in the liver of TXNIP-KO mice, but not of WT mice, after fasting for 24 h by H&E staining. By Oil Red O staining, there was no obvious difference in lipid accumulation between the WT and TXNIP-KO mouse liver in the fed state. In the fasted state, lipid droplets accumulated in the WT mouse liver to some extent, but accumulation of coarse lipid droplets was prominent in the TXNIP-KO mouse liver (Fig. 3).

We also assessed glycogen accumulation in the liver by periodic acid Schiff (PAS) staining (Fig. 3). Glycogen accumulation in the liver of TXNIP-KO mice was lower compared to that of WT mice in both the fed and fasted state, indicating that TXNIP-KO mice can utilize glycogen as a source of Glu and depend more heavily on Glu metabolism compared with WT mice. Our results also suggest that TXNIP-KO mice might have difficulties in lipid metabolism even in the fed state, leading to a lower level of glycogen accumulation.

### Changes in mitochondrial morphology and expression of mitochondria-related genes in the liver of TXNIP-KO mice

**Figure 4:**
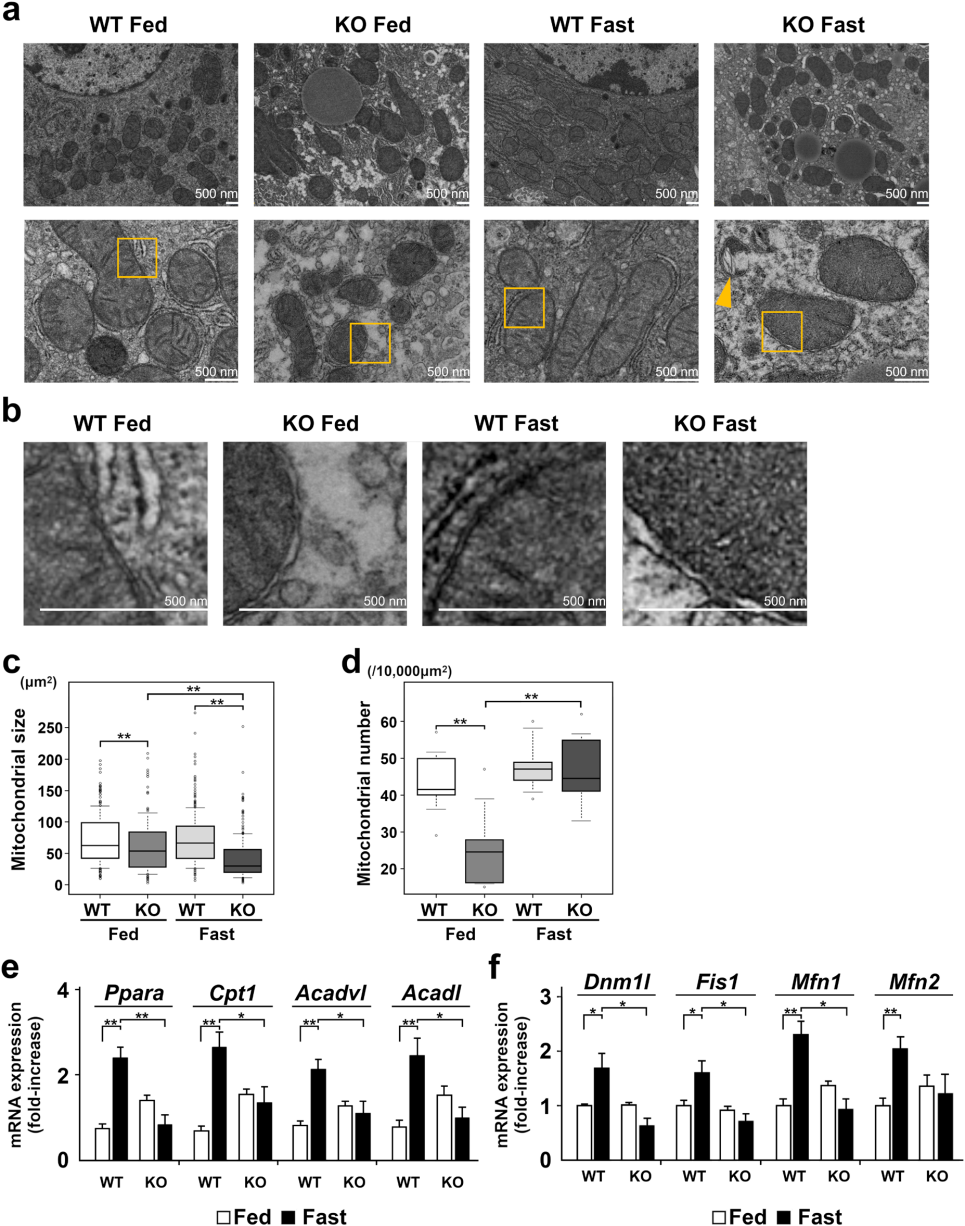
Morphology and mitochondrial β-oxidation are altered in TXNIP-KO mice during fasting. The morphology and the expression of β-oxidation-related and mitochondrial fission–fusion related genes, especially in the fasted state, were evaluated by TEM and RT-qPCR, respectively. (**a**) Top row: Mitochondrial size and number were compared. The size of mitochondria was small and the number of mitochondria increased in TXNIP-KO mice during fasting, although there appeared no obvious difference in the number of mitochondria between the both type of mice. Bottom row: Mitochondrial structures were compared. Although no obvious difference was found in the structure of cristae between WT and TXNIP-KO mice in the fed state, mitochondria were swollen and lacked cristae, and anomalously broken mitochondria (arrow head) were also observed in TXNIP-KO mice in the fasted state (scale bar = 500 nm). (**b**) The areas indicated by the boxes in figure (**a**) are enlarged. Compared with WT mice, the irregularity in the outer membrane was found in TXNIP-KO mice during fed state and this membrane irregularities were more evident during the fasted state. (**c**,**d**) Ten foci from each group were examined, and the mitochondrial size and number were quantitated. Mitochondrial size was statistically smaller in the liver of TXNIP-KO mice during the fed state, and the difference became more prominent during the fasted state, despite the fact that the number of mitochondria in the fasted state was not different between WT and TXNIP-KO mice. (**e**) Differences in the expression of β-oxidation-related genes or proteins between WT and TXNIP-KO mice. Overall, β-oxidation-related gene expression was upregulated in WT mice during the fasted state, but this change was not observed in TXNIP-KO mice. Expression of β-oxidation-related genes (Ppara, Cpta, Acadvl, and Acadl) was significantly higher in WT mice than in TXNIP-KO mice during the fasted state, indicating impaired β-oxidation in TXNIP-KO mice during the fasted state. (**f**) Gene expression of molecules related to mitochondrial fission and fusion (Dnm1l, Fis1, Mfn1, Mfn2) was examined. The expression of these molecules was upregulated in fasted state in WT mice, but this change was not found in TXNIP-KO mice. *p < 0.05. **p < 0.01 (in each group, six mice were used for RT-qPCR). *TEM* transmission electron microscopy, *TXNIP* thioredoxin-interacting protein, *WT* wild-type mice, *KO* knockout mice, *PPARα* peroxisome proliferator-activated receptor-α, *CPT1* carnitine palmitoyltransferase 1, *ACADVL* acyl-CoA dehydrogenase very long chain, *ACADL* acyl-CoA dehydrogenase long chain.

We next examined ultrastructural morphological changes in mitochondria by transmission electron microscopy (TEM). Under a low magnification (Fig. 4a, Top row), the liver of TXNIP-KO mice contained a number of small mitochondria in the fed state and the number of mitochondria appeared increased in the fasted state. The number of mitochondria appeared also increase in the liver of WT mice in the fasted state. Under a higher magnification (Fig. 4a, Bottom row), mitochondria in the liver of TXNIP-KO mice were morphologically aberrant with swelling and the absence of clear cristae, and anomalously broken mitochondria were also detectable (arrow head) in the fasted state. In the fed state, there was no obvious abnormality in the structure of cristae, but mitochondrial outer membranes were irregular in the liver of TXNIP-KO mice. This membrane irregularities were more evident during fasting (Fig. 4a bottom row, areas indicated by squares were further magnified in Fig. 4b). We quantitated the size and the number of mitochondria in the liver of WT and TXNIP-KO mice. The size of mitochondria was significantly smaller (*p* < 0.001) in the liver of TXNIP-KO mice in both fed and fasted state, and the number was also significantly lower (*p* < 0.001) in TXNIP-KO mice in the fed state (Fig. 4c,d).

During starvation, β-oxidation plays the major role in the generation of energy by oxidizing fatty acids, especially when Glu availability is low^29^. We examined the expression of genes related to β-oxidation, including peroxisome proliferator-activated receptor-α (PPARα), carnitine palmitoyltransferase 1 (CPT1), acyl-CoA dehydrogenase very long chain (ACADVL), and acyl-CoA dehydrogenase long chain (ACADL). Expression of all four genes significantly increased in the liver of WT mice in the fasted state. In the liver of fed TXNIP-KO mice, the expression of these genes appeared higher compared to the liver of WT mice but the expression significantly decreased after fasting (Fig. 4e). Thus, in the liver of fasted TXNIP-KO mice, mitochondrial damage was more severe and β-oxidation appeared impaired.

Damaged mitochondria split into small sizes by the process of mitochondrial fission, leading to autophagy. As shown above, mitochondrial size and number were smaller in the liver of TXNIP-KO mice, suggesting an impairment of mitochondrial fission and fusion in KO mice. Therefore we examined the mRNA level of several molecules that play an essential role in mitochondrial fission, fusion or ER-mitochondria contact, including dynamin 1-like protein (Dnm1l), mitochondrial fission protein 1 (Fis1), mitofusin 1 (Mfn1), and mitofusin 2 (Mfn2)^30,31^. In WT mice, the expression of all four mRNA was upregulated, but this change was not found in TXNIP-KO mice (Fig. 4f), suggesting that the regulation of mitochondrial fission and fusion is impaired in TXNIP-KO mice and partly explaining the reason why the number of small mitochondria increased in the liver of TXNIP-KO mice.

### Dysregulation of autophagy in the liver of TXNIP-KO mice

**Figure 5:**
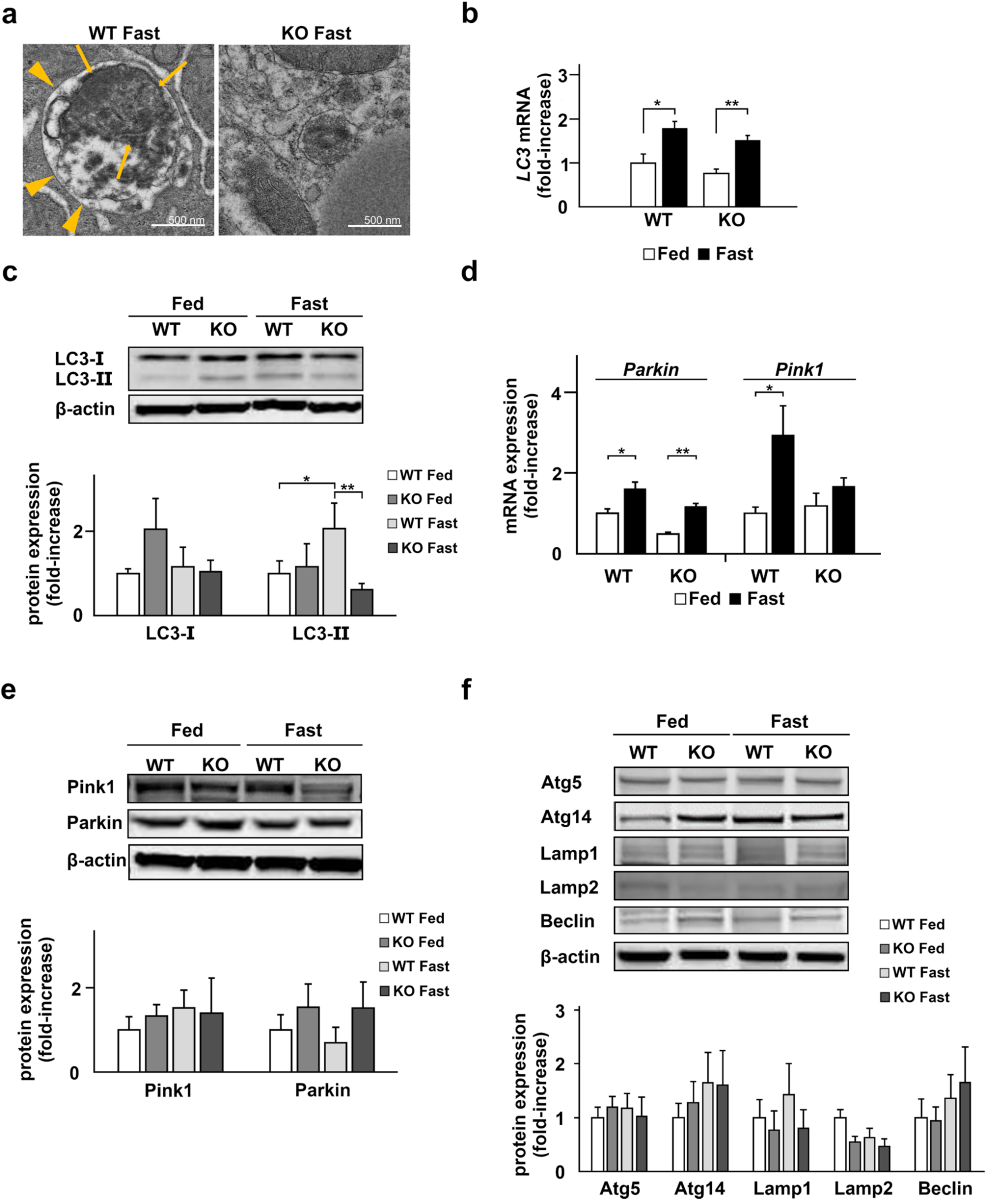
Autophagy is not activated in TXNIP-KO mice during fasting. Deficiency in autophagy was observed in the liver of TXNIP-KO mice. (**a**) The morphological differences of mitochondria between WT and TXNIP-KO mice were examined by TEM. In the liver of WT mice, autophagy increases during the fasted state, and in the present study, increased number of autophagosomes (arrow heads) containing mitochondria (arrows) were observed. By contrast, autophagosomes were rarely found in the liver of TXNIP-KO mice by TEM analysis. (**b**,**c**) The levels of LC3 mRNA and protein were evaluated by RT-qPCR and western blotting, respectively. The expression LC3 mRNA was upregulated during fasting in both WT and TXNIP-KO mice but there was no obvious difference between WT and KO mice. In WT mice the expression of LC3-II, a key molecule in autophagy, was upregulated during the fasted state, but this change was not observed in TXNIP-KO mice. The expression of LC3-II was statistically lower in TXNIP-KO mice during the fasted state. (**c**,**e**) The levels of Pink1 and Parkin, key molecules in mitophagy, were examined by RT-qPCR and western blotting, revealing that the expression patterns during the fed and fasted states were similar between WT and KO mice. (**f**) Other proteins playing essential roles in autophagy, Atg5, Atg14, Lamp1, Lamp2, and Beclin, were examined, and no statistical significance was found between WT and TXNIP-KO mice in the fed and fasted state in both strains. *p < 0.05, **p < 0.01 (five mice per strain were used for RT-qPCR, and ten were used for western blotting). *TXNIP* thioredoxin-interacting protein, *WT* wild-type mice, *KO* knockout mice, *WT* wild-type mice, *KO* knockout mice, *TEM* transmission electron microscopy, *LC3* microtubule-associated protein light chain 3, *Pink1* PTEN induced kinase 1, *Atg5* autophagy related 5, *Atg14* autophagy related 14, *Lamp1* lysosomal-associated membrane protein 1, *Lamp2* lysosomal-associated membrane protein 2.

To determine the underlying etiology causing the impaired clearance of damaged mitochondria in TXNIP-KO mice during the fasted state, we reexamined the structure of mitochondria more in detail by TEM. As shown in Fig. 5a, the number of autophagosomes (indicated by arrow heads) containing mitochondria (indicated by arrows) increased and damaged mitochondria were degraded in the liver of WT mice during the fasted state; however, autophagosomes were rarely found in the liver of TXNIP-KO mice during the fasted state (Fig. 5a), implying that dysregulation of autophagy was responsible for the accumulation of damaged mitochondria in the liver of TXNIP-KO mice.

Autophagy is a process eliminating disturbed macro-molecules or organelles by autophagosomes and LC3 plays a critical role in autophagy; LC3-II labels autophagosome membrane and promotes autophagy^32^. Though there was no statistical difference in the expression of LC3 mRNA between WT and TXNIP-KO mice, western blot (WB) analysis revealed that the level of LC3-II, the activated form of LC3, was significantly lower in the liver of TXNIP-KO mice than WT mice (*p* = 0.006) during fasting, indicating the decreased autophagy activity in the absence of TXNIP (Fig. 5b,c).

We next examined the level of Parkin and PTEN induced kinase 1 (Pink1) which play a role in mitophagy, autophagy specific to mitochondria. There was no obvious difference between WT and TXNIP-KO mice at both mRNA and protein expression levels (Fig. 5d,e).

Other proteins relating to autophagy, autophagy related 5 (Atg5), Atg14, lysosomal-associated membrane protein 1 (Lamp1), Lamp2, and Beclin, were also evaluated and no statistically significant difference was found between WT and TXNIP-KO mice (Fig. 5f).

### Dysregulation of ER stress and the UPR in TXNIP-KO mice

**Figure 6:**
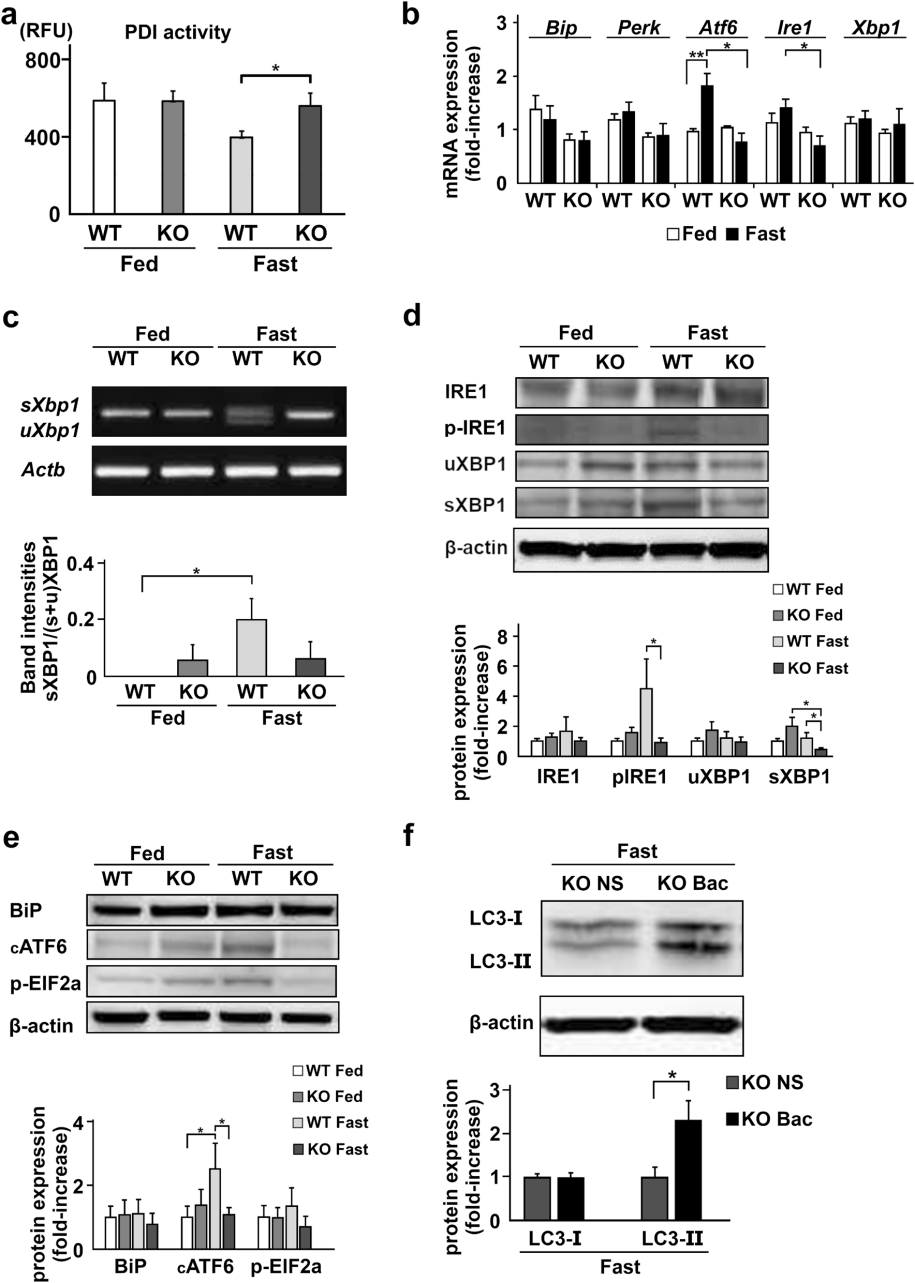
The ER stress-induced UPR and autophagy signaling during fasting are impaired in the liver of TXNIP-KO mice. (**a**) PDI activity was measured by the ProteoStat(R) PDI assay kit. PDI activity decreased during the fasted state in WT mice, but not in TXNIP-KO mice. (**b**) Differences in the expression of ER stress signaling-related genes between WT and TXNIP-KO mice were examined by RT-qPCR. In WT mice, expression of these genes increased in the fasted state, but this change was not observed in TXNIP-KO mice, and the expression of IRE1 mRNA, which is important in the induction of autophagy, was significantly lower in TXNIP-KO mice in the fasted state. (**c**) The expression of sXBP and uXBP1 mRNA was evaluated by PCR after RNA extraction and cDNA synthesis. Although upregulation of sXBP1 mRNA was found in WT mice during the fasted state, this upregulation was not found in TXNIP-KO mice. (**d**) Phosphorylation of IRE1 and the levels of uXBP1 and sXBP1 were examined by western blotting. Decreased phosphorylation of IRE1 and decreased level of sXBP1 were observed in TXNIP-KO mice, which implies decreased ER stress signaling for the induction of autophagy during the fasted state in TXNIP-KO mice. (**e**) Other proteins important for ER stress signaling, BiP, cATF6, and p-EIF2a, were also examined. Although the expression of these protein tended to be upregulated during the fasted state in WT mice, this tendency was not found in TXNIP-KO mice. Statistically significant differences were found in the expression of cATF6 between the fed and fasted states in WT mice, and WT and TXNIP-KO mice during fasted state. (**f**) Protein expression of LC3-II was restored in the fasted state when bacitracin was injected to the TXNIP-KO mice before 24-h fasting. *p < 0.05, **p < 0.01 (in each group, six mice were used for PDI analysis, RT-qPCR or PCR, and ten were used for western blotting). *UPR* unfolded protein response, *ER* endoplasmic reticulum, *TXNIP* thioredoxin-interacting protein, *PDI* protein disulfide isomerase, *BiP* binding immunoglobulin protein, *PERK* PKR-like endoplasmic reticulum kinase, *ATF6* activating transcription factor 6, *IRE1* inositol-requiring enzyme 1, *p-IRE1* phosphorylated IRE1, *sXBP1* X-box binding protein 1 sliced form, *uXBP1* XBP1 unsliced form, *cATF6* cleaved ATF6, *p-EIF2a* phosphorylated eukariotic initiation factor 2a, *WT* wild type mice, *KO* knockout mice, *NS* normal saline, *Bac* bacitracin.

Finally, we examined the signaling pathway that may lead to the autophagy in the liver of fasted mice, namely ER stress and the UPR. TXNIP was previously identified as a binding partner of PDI and shown to regulate the enzymatic activity of PDI^24^, suggesting that TXNIP acts in the upstream of the UPR and may affect the activity of PDI. As shown in Fig. 6a, PDI activity decreased in the fasted state in the liver of WT mice, but this change was not detected in the liver of TXNIP-KO mice. A statistically significant difference was noted in the PDI activity between WT and TXNIP-KO mice (*p* = 0.026) during the fasted state.

We next examined the expression of genes associated with the sensing and transmission of ER stress, such as the binding immunoglobulin protein (BiP), PERK, ATF6, IRE1 and XBP1 gene. The expression of PERK and XBP-1 mRNA did not show any difference between WT and TXNIP-KO mice in either the fed or the fasted state. The expression of ATF6 increased in the liver of WT mice in the fasted state, but this increase was not detected in the liver of TXNIP-KO mice. The expression of IRE1 mRNA was significantly lower in the liver of TXNIP-KO male mice compared with that of WT mice during the fasted state (Fig. 6b).

IRE1 is an enzyme with both kinase and RNase activity required for specific splicing of unspliced X-box binding protein 1 (uXBP1) mRNA to spliced form (sXBP1) of mRNA, and the phosphorylation of the activation loop is an important step in IRE1-mediated UPR activation^33^. IRE1-mediated XBP1 mRNA splicing is reported necessary for ER stress-induced autophagy in auditory cells^15^. Therefore, we evaluated the expression of sXBP1 mRNA variants by RT-PCR and revealed that despite the expression of sXBP1 was upregulated during the fasted state in WT mice, the expression of this variant was not detected in TXNIP-KO mice (Fig. 6c). Furthermore, we examined the level of phosphorylated IRE1 (p-IRE1) and sXBP1 in the liver of fasted mice by western blotting. The level of p-IRE1 and sXBP1 was elevated in the liver of WT mice during the fasted state, but the levels of these proteins were significantly lower in the liver of TXNIP-KO mice (Fig. 6d). We also examined the levels of other proteins associated with ER stress, including BiP, cleaved ATF6 (cATF6) and phosphorylated eukariotic initiation factor 2a (p-EIF2a) and found that although the level of cATF significantly increased in WT mice after fasting, this increase was not found in TXNIP-KO mice (Fig. 6e).

To confirm that the lack of decrease in PDI activity was responsible for the decreased LC3-II expression in the liver of TXNIP-KO mice, we treated TXNIP-KO mice with bacitracin, a widely used inhibitor of PDI. As shown in Fig. 6f, the level of LC3-II in the liver of TXNIP-KO mice during the fasted state was restored (*p* = 0.017) by bacitracin treatment before fasting (Fig. 6f). These data indicate that impaired ER stress-induced UPR activation, likely at the PDI level, leads to the dysregulation of autophagy in the liver of TXNIP-KO mice during fasting.

## Discussion

In the present manuscript, we examined the role of TXNIP in the ER stress, UPR, and autophagy after fasting in the mouse liver. TXNIP-KO male mice showed hyperlipidemia and LS in the fasted state, which is consistent with the findings reported previously^19^. Although mitochondrial deficiency was suspected in a previous study^19^, its exact mechanisms have not been demonstrated to date. We demonstrated the presence of mitochondrial deficiency in TXNIP-KO mice during the fasted state at a molecular and histological level by showing decreased expression of β-oxidation-related genes, and swollen mitochondria without cristae as observed by TEM. Furthermore, TEM analysis also showed the irregularity in the outer membrane of mitochondria, and mitochondrial disturbance was even seen in the fed state. Mitochondria are known to significantly influence LS and hyperlipidemia^26–28^. Normally, lipid droplets increase β-oxidation by interacting with mitochondria under low Glu conditions^27^, and energy sources are switched from Glu to lipids. Mitochondrial dysfunction is known to reduce β-oxidation, leading to hyperlipidemia^26^ and lipid accumulation in the liver^28^. Especially, increased TG levels during the fasted state in TXNIP-KO mice may indicate impaired β-oxidation because acyl-CoA is used in β-oxidation or TG production and thus, it is speculated that decreased β-oxidation might cause an increase in TG production. Taking these facts into account, damaged mitochondria are suspected to be the cause of LS and hyperlipidemia in the liver of TXNIP-KO mice during the fasted state. In the present study, despite that the serum ALT level were higher in the TXNIP-KO mice in fasted state, no statistical change was found between WT and TXNIP-KO mice. This suggest that the changes in TXNIP-KO mice were not sufficient to cause damages to liver tissues, but a longer fasting time may cause structural damage because past report showed a marked increase of ALT level after 48-h fasting^19^. It was interesting to find that female TXNIP-KO mice were more susceptible to fasting than male mice. This might be partially explained by higher insulin sensitivity in female mice as seen in human^34^, but additional studies are required to identify the exact mechanism for this observation.

Autophagy is a mechanism that removes stressed organelles or proteins. This mechanism does not operate well in the liver of TXNIP-KO mice during fasting. We demonstrated that morphologically aberrant mitochondria were prominent in TXNIP-KO mice and the size of mitochondria was smaller compared with that in WT mice as observed by TEM. It is known that disturbed mitochondria are split into small sizes before the process of autophagy and are captured by autophagosomes^35^. These facts imply that damaged and split mitochondria accumulate in the liver of TXNIP-KO mice during the fasted state. During autophagy, LC3-I is converted into LC3-II; LC3-II is essential for autophagosome formation^36^. In the present study, upregulation of TXNIP and an increase in LC3-II were seen in WT mice in the fasted state, but they were not observed in TXNIP-KO mice. Previously, it was demonstrated that TXNIP positively regulates autophagy^37^. From these observations, we speculate that TXNIP has a significant influence on autophagy, and damaged and split mitochondria cannot be cleared in TXNIP-KO mice in the fasted state because autophagy is not activated. We examined other molecules essential for autophagy or mitophagy. Overall, no significant difference was found between WT and TXNIP-KO mice after 24-h fasting. Many of these molecules tended to be upregulated in WT mice during fasted state, this tendency was not found in TXNIP-KO mice. It was also reported that TXNIP was able to influence mitophagy^38^. Additional studies are required to evaluate the contribution of these molecules. Additionally, our study revealed that the expression of fission- or fusion-related genes, including Mfn1, decreased during the fasted state in TXNIP-KO mice compared with WT mice, and this might also affect the downregulation of autophagy. Normally, damaged mitochondria split into small sizes by the process called fission and this process may be disturbed in TXNIP-KO mice. It is also known that autophagosomes are formed at the ER-mitochondria contact sites, and Mfn1 plays an important role in contacting ER and mitochondria^31^. Thus, disturbance in fission and mitochondria-ER contact may also result in decreased autophagy in TXNIP-KO mice during the fasted state.

ER stress is induced by various stresses such as fasting, viral infection, or inflammation and the UPR is provoked to resist^39–41^, although excessive ER stress causes cell death^12^. IRE1, PERK, and ATF6 are ER stress sensor proteins. ER stress signaling is initiated when unfolded proteins increase and activate these sensor proteins^42^. In particular, IRE1 plays important roles in cell survival and apoptosis, although the roles of IRE1 may be complex depending on the conditions^14,43^. It has been demonstrated that ER stress promotes autophagy via the IRE1-XBP1 signaling pathway^11,15,41,44^. Phosphorylation of IRE1 promotes the conversion of unspliced form of XBP1 (uXBP1) into spliced form of XBP1 (sXBP1), and sXBP1 induces autophagy by promoting the conversion of LC3-I into LC3-II^7,10,11,13,45^. We demonstrate that gene expression of ER stress sensor proteins and sXBP1 was suppressed and protein expression of p-IRE1 and sXBP1 was significantly decreased in the liver of TXNIP-KO mice compared with that in WT mice in the fasted state. These results imply a disturbance in the IRE1-XBP1 signaling pathway in TXNIP-KO mice. In addition, gene expression of ATF6 and protein expression of cATF6 were significantly lower in TXNIP-KO mice in fasted state and this is also important, because there is a report indicating autophagy is activated by the signal downstream of AFT6^46^. Although TXNIP has been suggested as a downstream protein of ER stress^47–49^, our results strongly suggest TXNIP to be one of the proteins regulating ER stress signaling in the mouse liver under fasted conditions.

The UPR is activated by decreased activation of chaperon proteins^50,51^. PDI works as the main chaperone protein^8,52^ and is involved in various diseases. PDI activity is decreased in diabetes and Alzheimer’s disease, leading to the accumulation of unfolded proteins in the ER, which triggers ER stress^9,39,53,54^. On the contrary, increased expression of PDI suppresses ER stress and supports cell survival or proliferation in cancer cells^9^. The fact that the expression of TXNIP is increased in diabetes and Alzheimer’s disease, and that the expression of TXNIP is decreased in tumor cells^55–57^ also implies a relationship between PDI and TXNIP. Furthermore, a previous report indicated that TXNIP directly interacts with PDI^24^. We measured PDI activity and observed that despite the decrease in PDI activity in the liver of WT mice after 24-h fasting, this change was not observed in the liver of TXNIP-KO mice. We also demonstrated the recovery of autophagy during the fasted state in TXNIP-KO mice after inhibition of PDI by bacitracin. Our data implied that TXNIP directly suppresses PDI activity in the liver during the fasted state. We attempted to detect direct interaction of TXNIP with PDI by immunoprecipitation, but our attempt was not successful. Despite these facts, there is a report showing that overexpressed TXNIP promotes PDI activity in HEK293 cells^24^. Furthermore, the absence of TXNIP may contribute to the improvement of acute kidney injury by promoting autophagy^58–60^. Considering these reports, the roles of TXNIP may differ depending on the conditions or organs.

In conclusion, TXNIP decreases PDI activity and promotes the ER stress signaling pathway that activates autophagy, contributing to the removal of damaged mitochondria during the fasted state in the mouse liver. As this process is impaired in TXNIP-KO mice, accumulation of damaged mitochondria occurs and lipid metabolism is disordered, leading to LS in the fasted state. Thus, TXNIP may serve as a new therapeutic target for the treatment of Reye-like syndrome, and deficiency in TXNIP may be associated with the mechanism of fatty liver caused by other clinical conditions, such as ALD or NAFLD, or drug-induced fatty liver.

## Methods

### Animals

C57BL/6 mice were purchased from CLEA Japan, Inc. (Tokyo, Japan) and used as WT mice. TXNIP-KO mice on a C57BL/6 background were generated by Dr. Junji Yodoi’s laboratory at Kyoto University and provided to us for this study^19^. All mice were maintained in a specific pathogen-free animal facility on a 12 h light/dark cycle at an ambient temperature of 21 °C and were given free access to water and food. All procedures involving animals were approved by the Animal Use Committee of Okayama University Graduate School of Medicine, Dentistry and Pharmaceutical Sciences (Approval OKU-2017586, OKU-2020037, OKU-2020722) and were performed in accordance with the National Institutes of Health Guidelines.

Seven-weeks-old WT and TXNIP-KO mice (male and female) were used in this study. Blood and liver of mice were examined before and after 24-h fasting. We also used bacitracin to suppress PDI activity in TXNIP-KO mice; bacitracin (0.1 ml, 100 mg/kg) or normal saline (0.1 ml) were given i.m. to TXNIP-KO mice and liver of mice were examined after 24-h fasting. This study is reported in accordance with ARRIVE guidelines as animals were injected with bacitracin.

### Kaplan–Meier analysis

Survival rates of WT or male or female TXNIP-KO mice were examined during fasting. When mice showed extremely declined activity and became moribund or lost 20% of the original weight, they were euthanized and treated as death.

### Blood analysis

Glu, TG, and TCH levels were measured using standard methods with a Dri-Chem 7000 V (FUJI FILM). Insulin levels were determined using an insulin assay kit (Morinaga Institute of Biological Science Inc., Tokyo, Japan). Serum free fatty acid (FFA) levels were measured using a LabAssay NEFA kit (Wako Pure Chemical Industries Inc., Osaka, Japan).

### Histological analysis

Livers were fixed in formalin and embedded in paraffin. Paraffin-embedded samples were sectioned at 5 µm and stained with hematoxylin and eosin (H&E) or PAS to evaluate glycogen accumulation. To evaluate lipid accumulation in the liver, livers were frozen in optimal cutting temperature compound (Sakura Finetek Japan Co., Ltd., Tokyo, Japan) and stained with oil red O following standard methods after being sectioned at 3 µm.

For transmission electron microscopy (TEM), liver samples were excised and fixed in a solution containing 2% glutaraldehyde and 2% paraformaldehyde, post-fixed in 2% osmium tetroxide, stained with aqueous uranyl acetate, dehydrated in a graded series of alcohol, and embedded in epoxy resin. Selected blocks were sliced into 80 nm ultrathin sections using an ultramicrotome (LEICA, EM UC7A, Wetzlar, Germany) and double-stained with uranyl acetate and lead citrate. These sections were examined using Hitachi H-7650 transmission electron microscope (Hitachi, Japan). The size of each mitochondrion was measured using Zeiss LSM Image Browser (Carl Zeiss, ver. 3.5). For evaluation of the number and size of mitochondria, 10 focused areas were evaluated for each sample by a staff who was not informed about the research.

### RT-qPCR

Total RNA was isolated from mouse liver using TRIzol reagent (Bioline Reagents Ltd., London, UK), and RNA concentration was determined using the NanoDrop spectrophotometer (Thermo Fisher Scientific). Complementary DNA (cDNA) was synthesized using the High-Capacity cDNA Reverse Transcription Kit (Thermo Scientific) with 800 ng of RNA for each sample. RT-qPCR was performed using StepOne Plus Real-Time PCR system with Taqman PCR master mix (Thermo Scientific). The primers used in this study were: TXNIP (Hs00197750_m1), Hspa5 (alias Bip) (Mm00517691_m1), Eif2ak3 (alias Perk) (Mm00438700_m1), Atf6 (Mm01295319_m1), Ern1 (alias is Ire1) (Mm00470233_m1), Xbp1 (Mm00457357_m1), Cpt1a (Mm01231183_m1), Ppara (Mm00440939_m1), Acadvl (Mm00444293_m1), Acadl (Mm00599660_m1), and β-actin (Mm02619580_g1) (Thermo Scientific). The expression levels of the genes of interest were normalized against those of β-actin.

### Detection of XBP1 splicing variants

After RNA extraction and cDNA synthesis as described above, the expression of sXBP and uXBP1 mRNA was evaluated by PCR. We used two primers, 5′-GATCCTGACGAGGTTCCAGA-3′ and 5′-ACAGGGTCCAACTTGTCCAG-3′. Two DNA products (sXBP1 and uXBP1) with 26-bp difference were amplified. Expression ratio of sXBP1 was calculated by sXBP1/(sXBP1 + uXBP1) after densitometrical analysis of PCR band intensities^61^. We also performed PCR and ACTIN was evaluated as a reference gene: we used primers, 5′-AGCCATGTACGTAGCCATCC-3′ and 5′-CTCTCAGCTGTGGTGGTGAA-3′.

### Western blotting

Mouse livers were lysed by radioimmunoprecipitation assay (RIPA) buffer containing 25 mM Tris–HCl pH 7.6, 150 mM NaCl, 1% NP-40, 1% sodium deoxycholate, and 0.1% sodium dodecyl sulfate–polyacrylamide (SDS). After homogenization, ultrasonic disintegration, and centrifugation, clear supernatants were collected. Protein concentrations in the supernatants were measured by BCA protein assay (Thermo Fisher Scientific). Equal amounts (50 μg) of protein in tissue lysates were subjected to SDS polyacrylamide gel electrophoresis (Thermo Scientific), and then transferred to nitrocellulose membranes. After blocking with Tris-buffered saline containing 0.1% Tween-20 (TBS-T) and 5% skim milk or bovine serum albumin, the membranes were incubated overnight with antibodies against TXNIP, Lamp1, Beclin, Atg14, XBP1 (Proteintech Group Inc.), LC3A/B, Lamp2, phosphorylated EIF2A, ATF6, β-actin (Cell Signaling Technology Inc.), IRE1, phosphorylated IRE1 (S724) (Abcam PLC), Pink1, Parkin (Novus Biologicals), or Atg5 (Sigma-Aldrich). After washing with TBS-T, the membranes were incubated with horseradish peroxidase-conjugated anti-rabbit IgG antibody (Cell Signaling Technology Inc.), and the target protein on the membrane was visualized using enhanced chemiluminescence system (ImmunoStar LD; Wako Pure Chemical Industries Inc.). Blots were photographed and analyzed using the Quantity One software (Bio-Rad Laboratories, Inc., ver. 4.4.).

### PDI activity assay

PDI activity was measured as follows. Liver tissue (100 mg) was lysed in 1 ml RIPA buffer without SDS and disintegrated by homogenization. After centrifugation at 10,000*g* for 10 min, supernatants were collected. The protein concentration was determined by BCA protein assay (Thermo Fisher Scientific), and equal amounts (1 mg) of protein samples were applied to the ProteoStat(R) PDI assay kit (Enzo Life Sciences Inc.). The fluorescent signals generated were detected by the multi-detection reader, FlexStation 3 (Sunnyvale, USA).

### Statistical analysis

Numerical variables were compared using the Student’s *t*-test. Laboratory data are reported as mean ± standard error. Statistical significance was set at *p* < 0.05. We used R commander (version 2.3-0) based on R (version 3.3.2, http://www.jichi.ac.jp/saitama-sct/SaitamaHP.files/ statmed.html) for statistical analyses.

## Supplementary Information

Supplementary Information.

## Data Availability

All data generated or analyzed during this study are included in this published article (and its Supplementary Information files).
