# Author Correction: Thioredoxin interacting protein protects mice from fasting induced liver steatosis by activating ER stress and its downstream signaling pathways

**DOI:** 10.1038/s41598-022-11823-3

**Published:** 2022-05-16

**Authors:** Hiroyuki Miyahara, Kosei Hasegawa, Masato Yashiro, Toshiaki Ohara, Masayoshi Fujisawa, Teizo Yoshimura, Akihiro Matsukawa, Hirokazu Tsukahara

**Affiliations:** 1 Department of Pediatrics, Okayama University Hospital, 2-5-1 Shikata-cho, Kita-ku, Okayama, 700-8558 Japan; 2 Department of Pathology and Experimental Medicine, Okayama University Graduate School of Medicine, Dentistry and Pharmaceutical Sciences, Okayama, Japan; 3 Department of Pediatrics, Okayama University Graduate School of Medicine, Dentistry and Pharmaceutical Sciences, Okayama, Japan

Correction to: *Scientific Reports* https://doi.org/10.1038/s41598-022-08791-z, published online 21 March 2022

The original version of this Article omitted an affiliation for Hiroyuki Miyahara. The correct affiliations are listed below.

Department of Pediatrics, Okayama University Hospital, 2‑5‑1 Shikata‑cho, Kita‑ku, Okayama 700‑8558, Japan.

Department of Pediatrics, Okayama University Graduate School of Medicine, Dentistry and Pharmaceutical Sciences, Okayama, Japan

The original Article and accompanying Supplementary Information file have been corrected.

