# Supplementary material for: Thioredoxin interacting protein protects mice from fasting induced liver steatosis by activating ER stress and its downstream signaling pathways
Source: Sci Rep. 2022 Mar 21;12:4819. doi: 10.1038/s41598-022-08791-z (PMC8938456; doi:10.1038/s41598-022-08791-z)
Supplement: Supplementary file 1 — Supplementary Information. [file 41598_2022_8791_MOESM1_ESM.pdf]

Supplementary Information (Original photos used for Figure 2, 5, and 6)

Thioredoxin interacting protein protects mice from fasting induced liver steatosis by activating ER stress and its downstream signaling pathways

Hiroyuki Miyahara, MD[1,3*], Kosei Hasegawa, MD, PhD[1], Masato Yashiro, MD, PhD[1], Toshiaki Ohara, MD, PhD[2], Masayoshi Fujisawa, MD, PhD[2], Teizo Yoshimura, MD, PhD[2], Akihiro Matsukawa, MD, PhD[2], Hirokazu Tsukahara, MD, PhD[3]

[1]Department of Pediatrics, Okayama University Hospital, Okayama, Japan

[2]Department of Pathology and Experimental Medicine, Okayama University Graduate School of Medicine, Dentistry and Pharmaceutical Sciences, Okayama, Japan

[3]Department of Pediatrics, Okayama University Graduate School of Medicine, Dentistry and Pharmaceutical Sciences, Okayama, Japan

Supplementary Figure S1

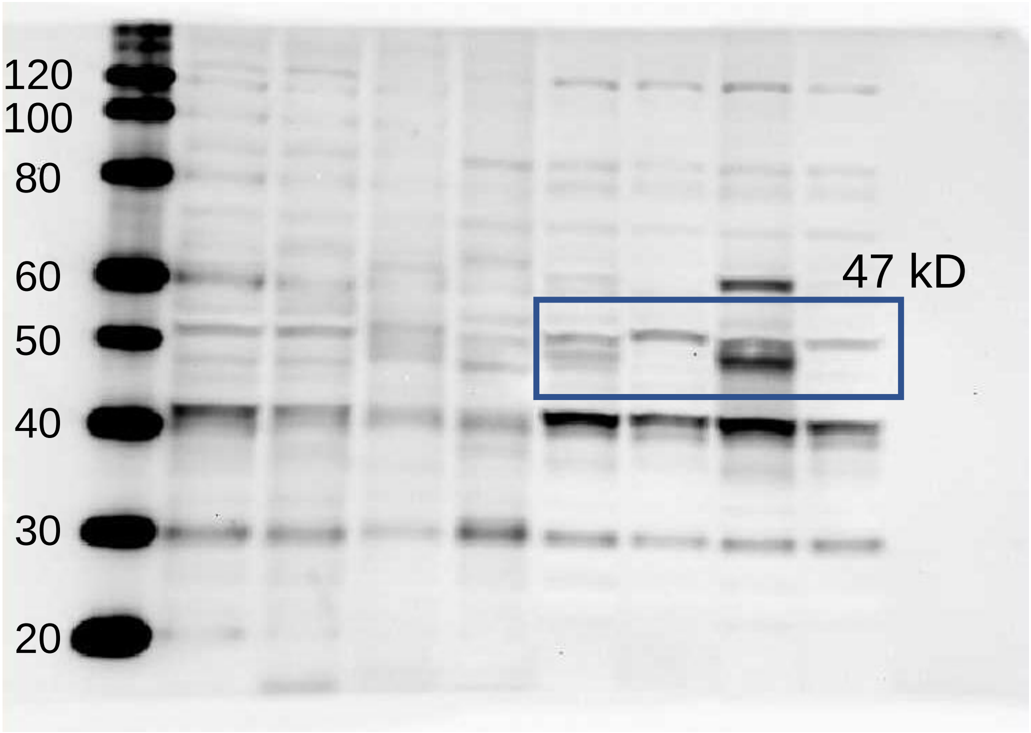

TXNIP

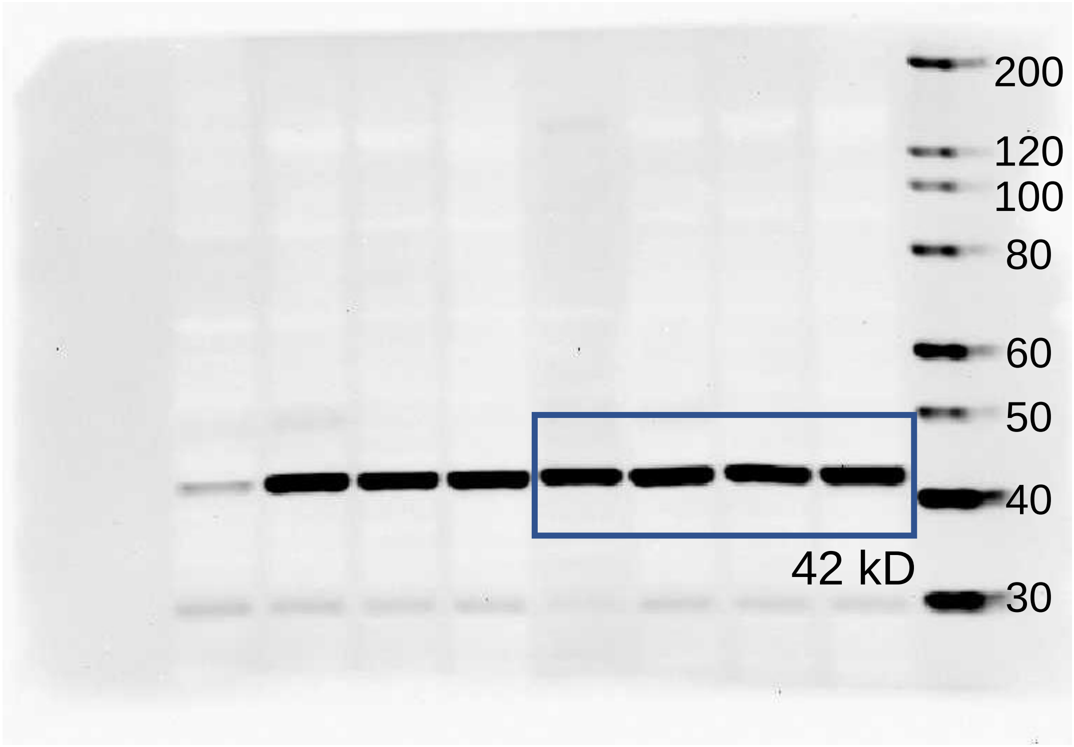

β-actin

Original photos used for Figure 2b. Western blotting of TXNIP and β-actin are shown. The areas indicated by the boxes in photos are used in Figure 2b. The MagicMark XP (Thermofisher) was used as a M.W. marker. TXNIP, thioredoxin-interacting protein.

Supplementary Figure S2

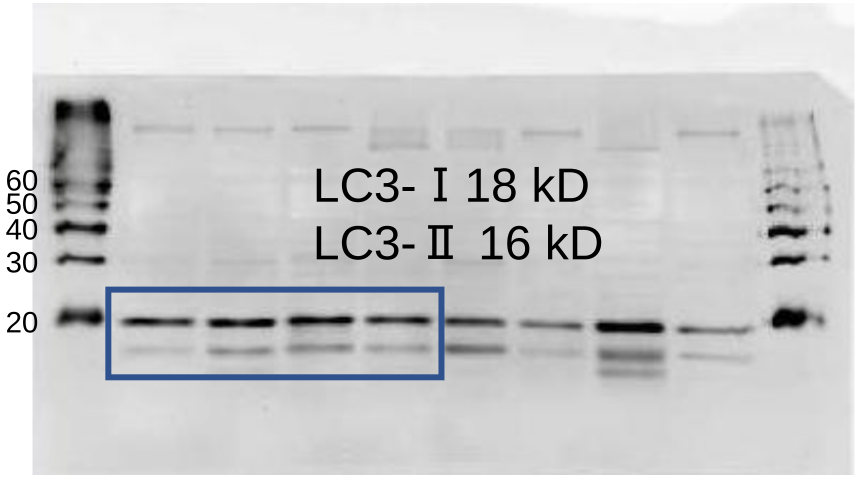

LC3

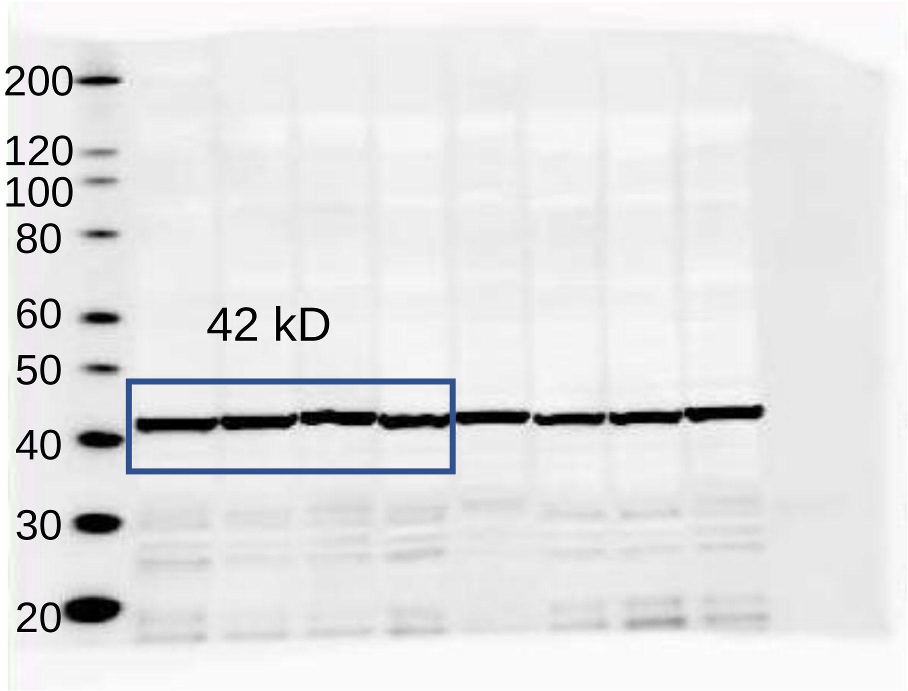

β-actin

Original photos used for Figure 5c. Western blotting of LC-3 and β-actin are shown. The areas indicated by the boxes in photos are used in Figure 5d. The MagicMark XP (Thermofisher) was used as a M.W. marker. LC3, microtubule-associated protein light chain 3.

Supplementary Figure S3

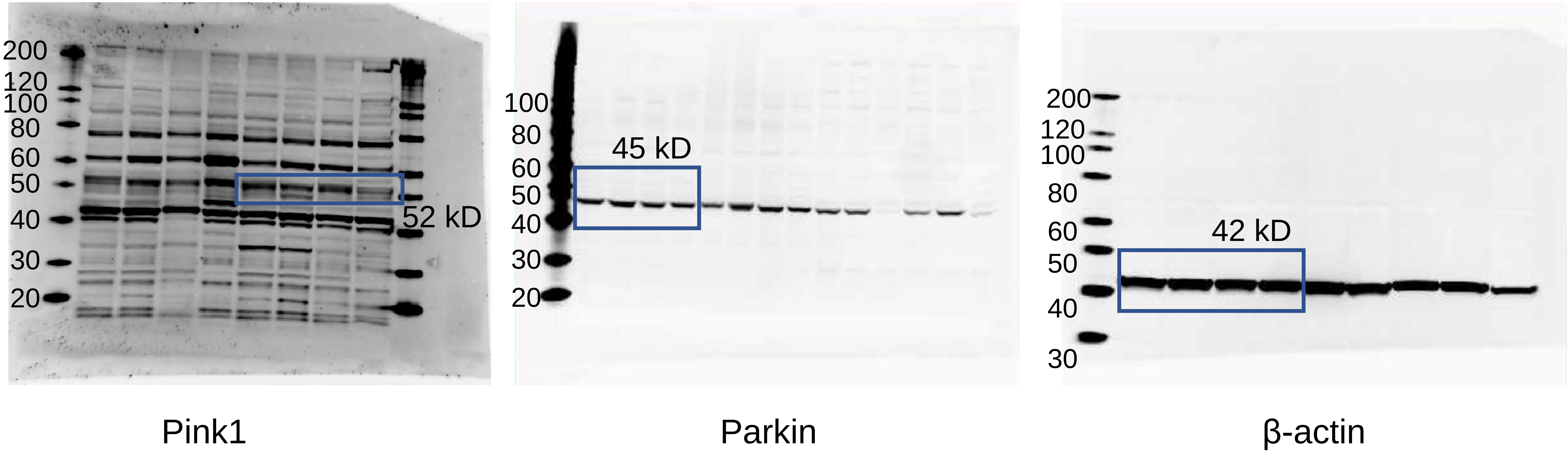

Original photos used for Figure 5e. Western blotting of Pink1, Parkin, and β-actin are shown. The areas indicated by the boxes in photos are used in Figure 5e. The MagicMark XP (Thermofisher) was used as a M.W. marker. PINK1, PTEN induced kinase 1

Supplementary Figure S4

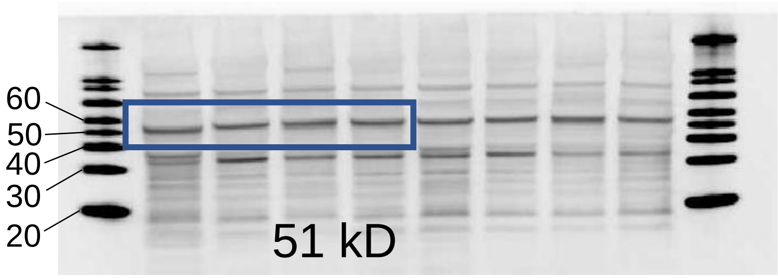

Atg5

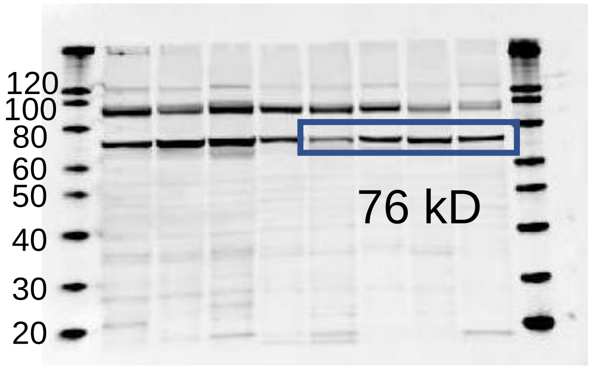

Atg14

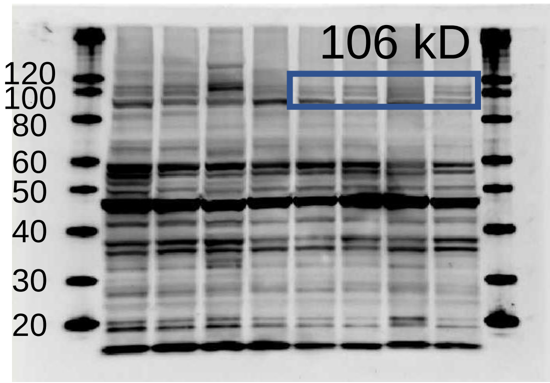

Lamp1

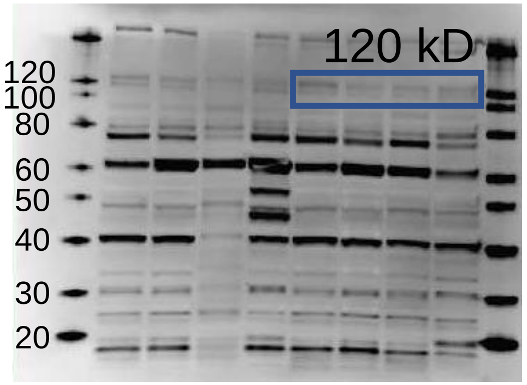

Lamp2

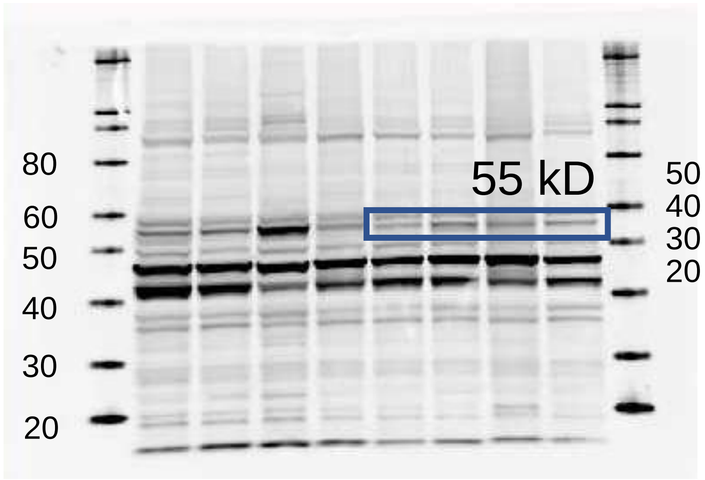

Beclin

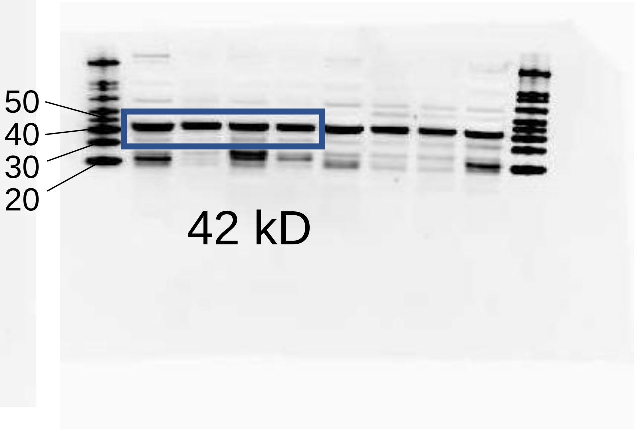

β-actin

Original photos used for Figure 5f. Western blotting of Atg5, Atg14, Lamp1, Lamp2, Beclin and β-actin are shown. The areas indicated by the boxes in photos are used in Figure 5f. The MagicMark XP (Thermofisher) was used as a M.W. marker. Atg5, autophagy protein 5; Atg14, autophagy-related protein 14; Lamp1, lysosomal-associated membrane protein 1;Lamp2, lysosomal-associated membrane protein 2

Supplementary Figure S5

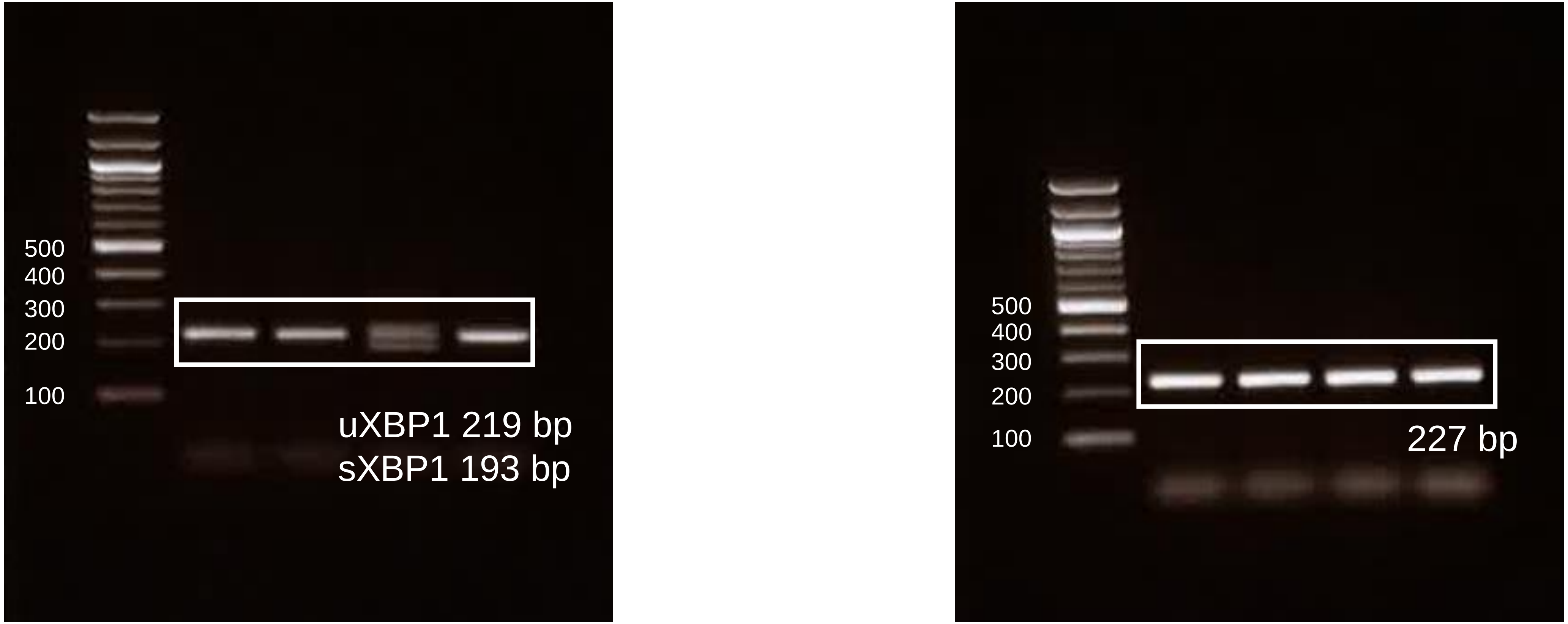

Original photos used for Figure 6c. The expression of sXBP, uXBP1 and β-actin mRNA was evaluated by agarose gel electrophoresis using PCR products after RNA extraction and cDNA synthesis. The areas indicated by the boxes in photos are used in Figure 6c. The 100 bp DNA Ladder (NEW ENGLAND Bio Labs) was used as a M.W. marker. sXBP1, X-box binding protein 1 sliced form; uXBP1, XBP1 unsliced form

Supplementary Figure S6

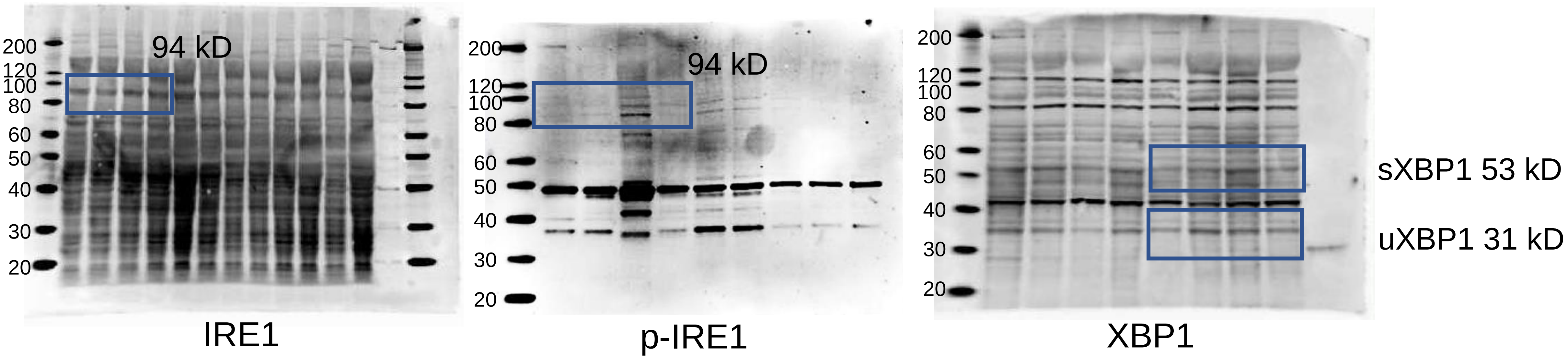

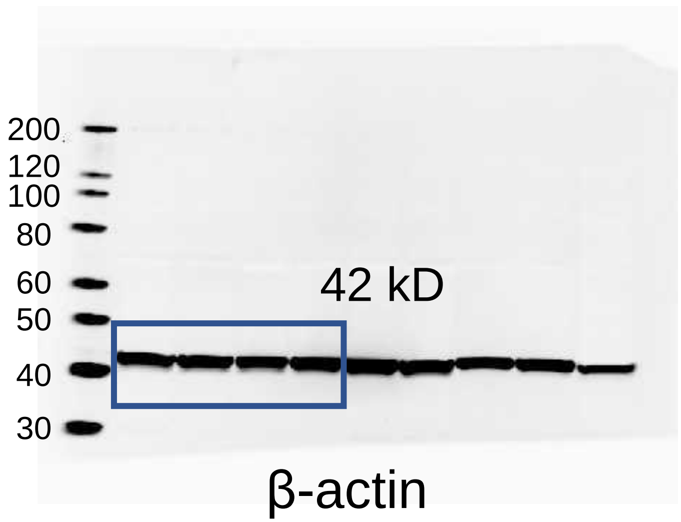

Original photos used for Figure 6d. Western blotting of IRE1, p-IRE1, XBP1 and β-actin are shown. The areas indicated by the boxes in photos are used in Figure 6d. The MagicMark XP (Thermofisher) was used as a M.W. marker. IRE1, inositol-requiring enzyme 1; p-IRE1, phosphorylated IRE1; XBP1, X-box binding protein 1; sXBP1, X-box binding protein 1 sliced form; uXBP1, XBP1 unsliced form

Supplementary Figure S7

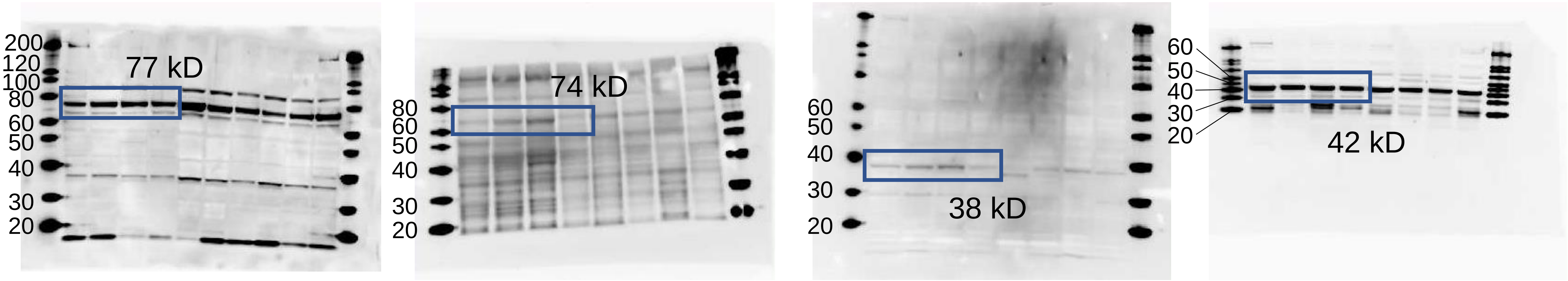

BiP cATF6 p-EIF2A β-actin

Original photos used for Figure 6e. Western blotting of BiP, cATF6, p-EIF2a and β-actin are shown. The areas indicated by the boxes in photos are used in Figure 6e. The MagicMark XP (Thermofisher) was used as a M.W. marker. BiP, binding immunoglobulin protein; p-EIF2a, phosphorylated eukariotic initiation factor 2a; cATF6, cleaved activating transcription factor 6.

Supplementary Figure S8

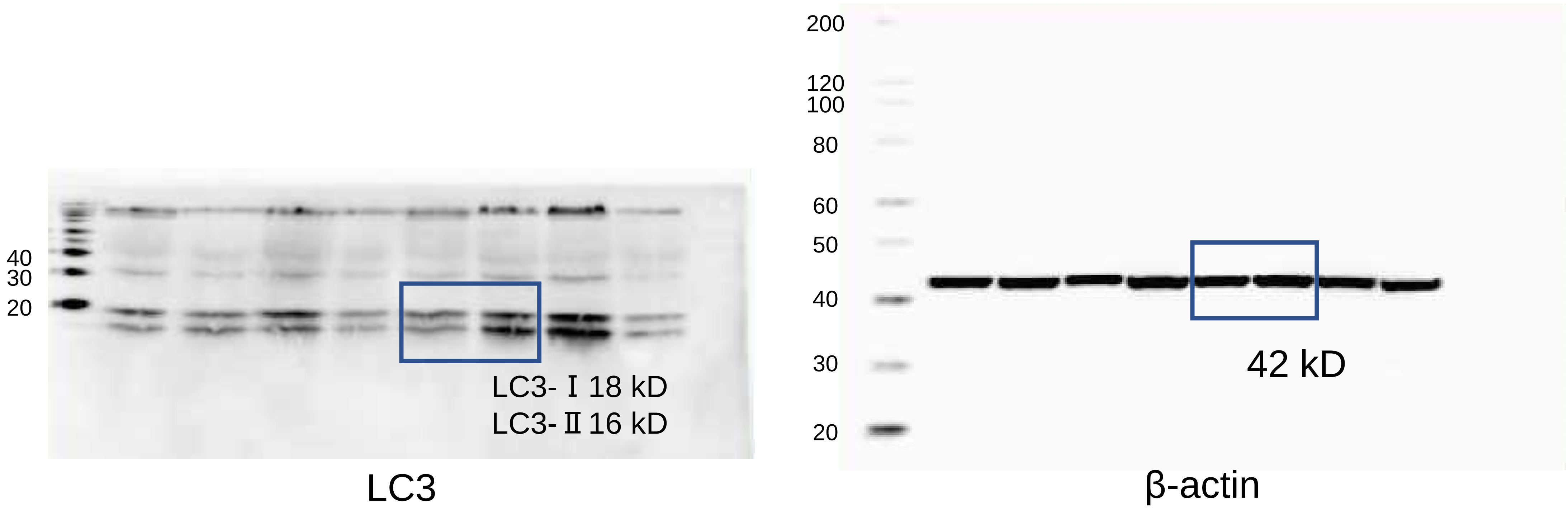

Original photos used for Figure 6f. Western blotting of LC-3 and β-actin are shown. The areas indicated by the boxes in photos are used in Figure 6f. The MagicMark XP (Thermofisher) was used as a M.W. marker. LC3, microtubule-associated protein light chain 3.
